# Amino–MIL-101(Fe)/Chitosan–Graphene Oxide Cross-Linked Nanocomposite for High-Performance Adsorptive Remediation of Wastewater Microplastics from Environmental Samples

**DOI:** 10.3390/polym18070878

**Published:** 2026-04-02

**Authors:** Amr A. Yakout, Ahmed S. Badr El-din, Amani Al Solami, Abeer H. Aljadaani

**Affiliations:** 1 Department of Chemistry, College of Science, University of Jeddah, P.O. Box 80237, Jeddah 21589, Saudi Arabia; 2 Department of Chemistry, Faculty of Science, University of Tabuk, Tabuk 71491, Saudi Arabia

**Keywords:** graphene oxide, chitosan, metal–organic framework (MOF), microplastics, adsorption

## Abstract

One of the main sources of microplastic pollution in aquatic ecosystems is municipal wastewater, and preserving the ecological security of water depends on its effective removal. In this study, a potential multi-functionalized nanocomposite (NH_2_-MIL-101(Fe)/CS/GO), which consists of an iron-based metal–organic framework (NH_2_-MIL-101(Fe)) integrated with chitosan (CS) as a biopolymer matrix and graphene oxide (GO) as a conductive support, was exploited to enhance microplastic removal via different adsorptive hydrophilic/hydrophobic interactions. According to adsorption tests, the removal efficiencies of NH_2_-MIL-101(Fe)/CS/GO for polyethylene terephthalate (PET) and polystyrene (PS) microplastics (25–30 μm) were 93.8% and 89.7%, respectively, at pH 6.2 and for 40 min of contact time. Adsorption isotherms were well fitted to both the Langmuir and the Freundlich models, and the maximum adsorption capacities of PET and PS were 321.4 and 255.1 mg·g^−1^, respectively. The removal efficiency reached 92.5% after six cycles. The proposed MOF-based CS/GO nanocomposite provides an efficient and durable method of controlling microplastic contamination in urban wastewater. The developed multi-functionalized nanocomposite offers excellent electrostatic and hydrophobic synergy through a large surface area and π–π interactions for GO, positively charged CS, and a very high surface area with tunable porosity for the amino–MIL-101 (Fe) moiety. The proposed MOF-based nanocomposite provides an effective and persistent method of reducing microplastic contamination in constructed wetlands and water/wastewater treatment plants.

## 1. Introduction

Microplastics (MPs), which pose a hazard to aquatic and human life, have become a common environmental contaminant due to poor plastic waste management [1,2]. MPs frequently enter aquatic environments through many sources, including rainwater, rivers, industrial discharges, storm drains, wind, tides, floods, and sewage disposal [3,4]. Globally, rivers, lakes, and oceans contain extensive amounts of MPs, which are plastic pieces with sizes less than 5 mm in diameter [5,6,7].

They are very common in urban water, especially in areas with a high population density [8]. Because of their exceptional persistence in aquatic habitats, microplastics present serious ecological hazards to water ecosystems, threatening the existence and well-being of aquatic life [9,10]. Additionally, microplastics can enter vital human physiological systems, such as the brain, heart, placenta, and circulation, which may result in neurological and cardiovascular disorders [11,12]. Furthermore, microplastics can harm microbial ecosystems and disrupt gene transfer pathways [13].

Microplastics are categorized into primary microplastics and secondary microplastics. Primary microplastics are microscopic in size and enter aquatic life from personal care products, e.g., cosmetics and facial scrubbers. Secondary microplastics result from the degradation of larger plastic debris by sunlight, water, wind, and other environmental factors. Daily human activities are the primary sources of microplastics in wastewater, including synthetic textile laundering, personal care products, and different domestic/industrial activities. Laundering accounts for the highest percentage of all primary microplastics in domestic wastewater, as the washing of synthetic clothing (acrylic, nylon, polyester and polyamide) releases millions of microfibers per wash cycle, which are then transported to wastewater treatment plants. Therefore, domestic wastewater is the main source through which microplastics enter water bodies [14]. A critical problem that should be addressed right away is preventing microplastics from migrating from urban household wastewater into aquatic environments [15,16].

Chemical removal, biological degradation, and physical removal are the three methods used to manage microplastics in aquatic environments. Chemical removal primarily involves photocatalysis and sophisticated oxidation processes [17], while adsorption, coagulation, and membrane filtration are examples of physical removal methods. While coagulation is quite effective at separating microplastics, the improper disposal of the flocs that are created might cause a secondary environmental release of microplastics [18]. Although membrane filtration is significantly more efficient than coagulation and adsorption when eliminating plastic, the fouling of the membrane, degradation, and high management and maintenance costs make it challenging in practical applications [19]. Nonetheless, the safety of the degraded byproducts remains a major concern when microplastics and debris from plastics are broken down using modern oxidation and photocatalysis techniques [18]. Microplastics cannot be quickly and effectively eliminated by biological degradation processes due to their inefficiency and long breakdown timeframes [20]. In general, technologies for physical treatment, especially adsorption, are reasonably advanced and exhibit a high level of microplastic removal effectiveness, making them viable, eco-friendly, and attractive substitutes for microplastic management and treatment.

The removal of microplastics using a variety of adsorbents, including metal–organic frameworks (MOFs), aerogels, carbon nanotubes, sponges, activated carbon, and biochar, has been thoroughly studied by researchers [21,22,23,24,25]. According to previously reported studies, carbon materials have an attractive effect on microplastics and can interact with them through functional groups and “sticking/trapping/entangling”. Such interactions either sequester microplastics inside porous structures or bind them onto carbonaceous materials, resulting in excellent removal efficacy [26,27]. However, this method is still challenging for separating and regenerating carbon-based molecules.

There is much published research using chitosan (CS), a biobased adsorbent, for the recovery of MPs. Because of its high surface area and cationic characteristics, which can be altered for a variety of derivatives with different solubility, biodegradability, biocompatibility, and selectivity toward pollutants, CS and its functionalized composites have drawn a lot of interest for capturing MPs and other multiple water pollutants at ambient pH [28,29].

Metal–organic frameworks (MOFs) are crystalline hybrid porous coordination polymers that are employed in several studies for microplastic remediation. The addition of functional groups (-NH_2_, -OH, -H, -Br, -NO_2_) to MOFs results in additional core and activation sites for improved adsorption of microplastics. This enhancement in MP removal performance was attributed to the synergistic effect of intensified hydrophobic and H-bonding interactions, emphasizing superior adsorption selectivity [30].

As such, MOFs are now considered to be one of the most important classes of materials for microplastic removal, but their practical applications are constrained by sensitivity to acidic conditions and moisture, and difficulties with separation and recovery, thus rendering them unstable in aqueous media [31]. These restrictions are addressed by immobilizing MOFs onto superhydrophobic auxiliary materials such as aerogels or sponges, thereby fabricating composites that exhibit high MP removal efficiencies. For example, a 90% MP removal capacity was achieved by MOF-based melamine and urethane and sponges [32,33,34]. However, it is essential to point out that sponge-type materials are essentially foamed plastics, and their degraded products could cause potential health and environmental hazards. In addition, these materials could also release their degradation products into the environment during microplastic removal, thereby causing secondary pollution. Moreover, the materials’ applications for microplastics are somewhat limited, and they are mainly used for adsorption applications on the laboratory scale. Thus, the development of practical, efficient, low-cost, and reusable nanosorbents is needed for the high-performance remediation of microplastic contamination in wastewater.

To achieve the efficient, green, and low-cost removal of PET/PS-MPs from wastewater, an MOF-biobased adsorption platform nanocomposite (NH_2_-MIL-101(Fe)/chitosan/graphene oxide, denoted as NH_2_-MIL-101(Fe)/CS/GO) was constructed using a green approach for efficient microplastic adsorption in different environmental samples. GO was chosen because it disperses well in water, has a large surface area, and carries oxygenated groups that can interact effectively with microplastics. NH_2_-MIL-101(Fe) was selected for its high porosity, large surface area, and amino groups, which provide additional adsorption sites and improve affinity toward the target particles. Together with chitosan, these components form a synergistic nanocomposite in which GO improves dispersion, the MOF supplies accessible active sites, and CS supports adsorption and structural integrity. HRTEM, SEM, EDX, BET, FTIR, XRD, and XPS investigations were used to systematically assess the surface morphology and chemical characteristics of the synthesized nanocomposite.

Extreme-pH and high-salinity conditions were used to evaluate stability and durability. Batch adsorption studies were used to assess microplastic removal performance. To produce stable, regenerable, highly effective, and pollution-free adsorbents for microplastic cleanup in aquatic environments, our effort combines theoretical understanding with experimental support.

## 2. Materials and Methods

### 2.1. Materials

All the chemicals were used as received, unless otherwise specified, and were of analytical reagent grade. Anhydrous ferric chloride (FeCl_3_), 2-aminoterephthalic acid (NH_2_–H_2_BDC), N,N-dimethylformamide (DMF), acetonitrile (ACN), acetic acid (purity ≥ 99.5%), anhydrous ethanol (purity ≥ 99.9%), calcium chloride dihydrate (CaCl_2_⋅2H_2_O), hydrochloric acid (HCl, 36%), sodium hydroxide (NaOH), and chitosan (CS; 200–600 mPa-s, 0.5% in 0.5% acetic acid at 20 °C) were acquired from Sigma-Aldrich (St. Louis, MO, USA). The supplier of graphene oxide (GO) powder was Sinopharm Chemical Reagent Co., Ltd. (Shanghai, China). We obtained magnesium chloride hexahydrate (MgCl_2_⋅6H_2_O) and sodium sulfate (Na_2_SO_4_) from Fluka Chemical (Buchs, Switzerland). Commercial microplastic powders, including polyethylene terephthalate (PET 500, mean diameter = 28 ± 2 µm) and polystyrene (PS MPs, mean diameter = 25 ± 3 µm), were purchased from Arabian Plastic Industrial Company (APICO, Riyadh, Saudi Arabia).

### 2.2. Preparation of NH_2_-MIL-101(Fe)/CS/GO Nanocomposite

NH_2_-MIL-101(Fe)/CS/GO was synthesized by a two-step method including the solvothermal growth of an amino-functionalized NH_2_-MIL-101(Fe), followed by the functionalization of the cross-linked CS-GO by the synthesized amino–MOF via blending in a ball-mill reactor. NH_2_-MIL-101(Fe) was prepared through a solvothermal reaction involving Fe(III) as a metal source and NH_2_-H_2_BDC as an organic linker [35,36,37]. In a representative synthesis, FeCl_3_·6H_2_O (10.0 mmol, 2.70 g) and NH_2_-H_2_BDC (5.0 mmol, 0.91 g) were dissolved under continuous stirring in DMF (60 mL) and deionized water (10 mL) to give a uniform precursor solution. HCl (37 wt%, 0.5–1.0 mL) was added as a mineral-acid modulator, if needed, to improve crystallization. The combined solution was then transferred to a Teflon-lined stainless-steel autoclave and heated at 150 °C for 12 h. After cooling naturally, the solid was collected by centrifugation, washed several times with DMF and ethanol, and then underwent solvent exchange with ethanol to extract residual guests. The product was dried and activated under vacuum at 120–150 °C to yield a porous NH_2_-MIL-101(Fe). CS was cross-linked onto GO sheets using 1.0 mL of glutaraldehyde, followed by functionalization with NH_2_-MIL-101(Fe). The composite was prepared at a mass ratio of 20% MOF: 50% CS: 100% GO through mechanical blending in a stainless-steel ball-mill reactor operated at 30 Hz for 20 min.

### 2.3. Characterization Techniques

Surface area and pore size distribution of the textural properties were calculated from N_2_ adsorption–desorption isotherms on a Quantachrome NOVA 4200e instrument (Boynton Beach, FL, USA). The concentration of PET and PS MPs was quantified by a turbidimeter (Hach 2100Q/2100Q, Loveland, CO, USA). The SEM (JEOL JSM-6010LV, Tokyo, Japan) and HRTEM (JEOL JEM-2100V) were employed to analyze the morphology and microstructure of the NH_2_-MIL-101(Fe)/CS/GO nanocomposite. Oxidation states and elemental composition of the surface were investigated by XPS (Thermo ESCALAB 250Xi, Waltham, MA, USA). Crystallinity and phase structure were analyzed with the aid of powder X-ray diffraction (XRD) (Rigaku D/MAX-2550, Tokyo, Japan) using Cu Kα radiation. The FT-IR spectra (Nicolet 400) were obtained in the range of 4000–400 cm^−1^ to prove the presence of functional groups. Zeta potential analysis was performed on a 1.0 wt% aqueous suspension using Zetasizer Nano ZS90 (Malvern, Malvern, UK). The pH of the solution was determined by a calibrated pH meter (Model 810; Fisher Scientific, Waltham, MA, USA) with a combined glass electrode.

### 2.4. PET/PS-MPs Batch Adsorption Study, Regeneration and Application Tests

For adsorption experiments, 100 mg of NH_2_-MIL-101(Fe)/CS/GO was added to 10 mL of PER or PS microplastic suspensions. To reach adsorption equilibrium, the mixtures were shaken using a thermostatic shaker at 250 rpm for 40 min at 25 °C. A turbidity meter was used to test the supernatant’s turbidity after filtering. Turbidity was selected since it has been shown to be more sensitive than the absorbance approach. Additionally, several turbidity calibration curves were created, all of which had *R*^2^ values greater than 0.995. Every experiment was carried out in triplicate, and data analysis and graphing were done using computed averages. NH_2_-MIL-101(Fe)/CS/GO was produced by drying used MOF-functionalized nanocomposites containing adsorbed PET or PS-MPs for 12 h to evaluate reusability. The applied nanocomposite had been immersed in 20 mL of ethanol, and then it was shaken at 250 rpm for 40 min at 25 °C to complete the desorption process. The regenerated adsorbent was utilized again in later adsorption tests once it had dried. The removal efficacy before and after regeneration was evaluated, and the adsorption–desorption cycle was repeated multiple times. PER/PS-MP removal efficiency and adsorption capacity were calculated using Equations (1) and (2), respectively:

(1) $$\%\;R=\;\;\frac{C_{o}-\;C_{t}\;}{c_{o}}\;\;\times \;\;100$$

(2) $$\%\;q_{t}=\;\frac{{(C}_{o}-C_{t})\;V\;}{m}\;\;$$

where *C_o_* and *C_t_* (mg L^−1^) are the initial and time-*t* MP concentrations, *V*(L) is the solution volume, and *m*(g) is the nanocomposite mass. All adsorption tests were conducted three times, and the average values were reported. The precision and accuracy of the method were evaluated through replicate determinations (recovery and RSD).

The removal effectiveness of PET/PS-MPs (15 mg L^−1^ solutions) by the NH_2_-MIL-101(Fe)/CS/GO nanocomposite was examined in relation to pH, salinity, and competitive ions. Solutions were adjusted between 2 and 10 for pH testing using either NaOH or 0.1 M HCl. Competitive ion tests were carried out using cations (Ca^2+^, Mg^2+^, Na^+^, and K^+^) and anions (SO_4_^2−^, NO_3_^−^, and Cl^−^), each at a concentration of 20 mM. Salinity experiments were carried out using NaCl concentrations ranging from 5 to 500 mM while keeping pH at 6.2. To evaluate the adsorption performance stability and reusability of NH_2_-MIL-101(Fe)/CS/GO for PET-MP adsorption, adsorption–desorption experiments were conducted under equilibrium conditions at 25 °C and 200 rpm for 40 min for six consecutive cycles in triplicate. Ethanol was used for regeneration and desorption of PET-MPs from the nanocomposite surface.

To evaluate the applicability of the NH_2_-MIL-101(Fe)/CS/GO nanocomposite in environmental water, wastewater, and MQ water samples, two samples of domestic wastewater, tap water, river water, and seawater taken from Egypt’s Mediterranean Sea between August and October 2025 were used in PET/PS-MP adsorption tests. The tap water sample came from an Egyptian laboratory tap at Alexandria University. River water was gathered from Egypt’s Nile River. All real water samples were filtered through 0.22 µm membrane filters and stored at 4.0 °C until use in experiments. The water quality parameters of the water samples are summarized in Table 1, including pH, total dissolved solids (TDS), and major anion/cation concentrations. Domestic, tap, and river water samples were tested without dilution for ion chromatography analysis, while ocean samples were diluted at a ratio of 1:1000 prior to measurement.

## 3. Results and Discussion

### 3.1. Characterization of NH_2_-MIL-101(Fe)/CS/GO

**Figure 1:**
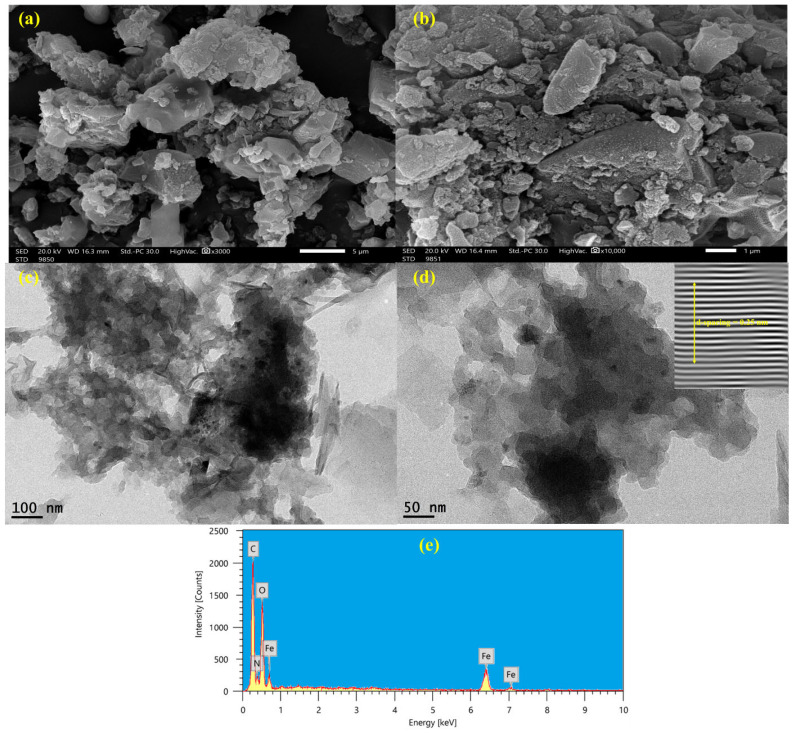
SEM (**a**,**b**) and HRTEM (**c**,**d**) images at different magnifications and EDX (**e**) of the NH_2_-MIL-101(Fe)/CS/GO.

Figure 1a,b display SEM images of the NH_2_-MIL-101(Fe)/CS/GO nanocomposite at different magnifications. The microscopic structure displays dispersed, linked aggregates and a porous, rough surface that are typical of the morphology of the hybrid composite. Fine dispersion in the two images suggests a good degree of integration between the amino-functionalized MOF, CS, and GO, which are essential for promoting the MPs’ adsorption. The nanocomposite structure allows a high surface area, with numerous active sites for the removal of MPs. The HRTEM images (Figure 1c,d) further confirm the nanostructure of the nanocomposite. A dense network of nanoparticles with varying sizes confirms the successful incorporation of NH_2_-MIL-101(Fe) into the CS/GO matrix, as displayed in Figure 1c. The presence of Fe-containing nanoparticles of the MOF in a well-defined crystalline nanocomposite is obvious in Figure 1d. The d-spacing of Fe nanoparticles was approximately 0.29 nm, corresponding to the (110) plane of Fe-based MOFs, confirming the crystalline nature and stability of NH_2_-MIL-101(Fe) in the functionalized nanocomposite. The EDX spectrum showed the fundamental elements of the NH_2_-MIL-101(Fe), GO, and CS, which are Fe, C, O, and N (Figure 1e). The C and N peaks show the presence of CS and GO, and the notable peak for Fe verifies the effective incorporation of Fe-based MOFs into the nanocomposite. The overall findings from the SEM, HRTEM, and EDX investigations show that the components are uniformly distributed and that the NH_2_-MIL-101(Fe)/CS/GO nanocomposite is structurally sound, making it a viable material for adsorption and environmental remediation applications. The tertiary nanocomposite exhibits a high specific surface area of 913 m^2^ g^−1^ and a total pore volume of 3.11 cm^3^ g^−1^, confirming its highly porous structure. In addition, the pore size distribution was analyzed and found to fall predominantly within the mesoporous range, which is consistent with the incorporation of CS and GO into the MOF. This hierarchical porosity is particularly advantageous for adsorption processes involving relatively large species. Instead, adsorption is expected to occur mainly on the external surface, at pore entrances, and within mesoporous regions where accessibility is not sterically restricted. Moreover, the high surface area and large pore volume provide abundant active sites and facilitate mass transfer, which enhances the interaction between the nanocomposite and microplastic particles. The presence of mesopores also reduces diffusion limitations and allows effective capture of nanoscale microplastics through surface adsorption, electrostatic interactions, and possible bridging mechanisms.

**Figure 2:**
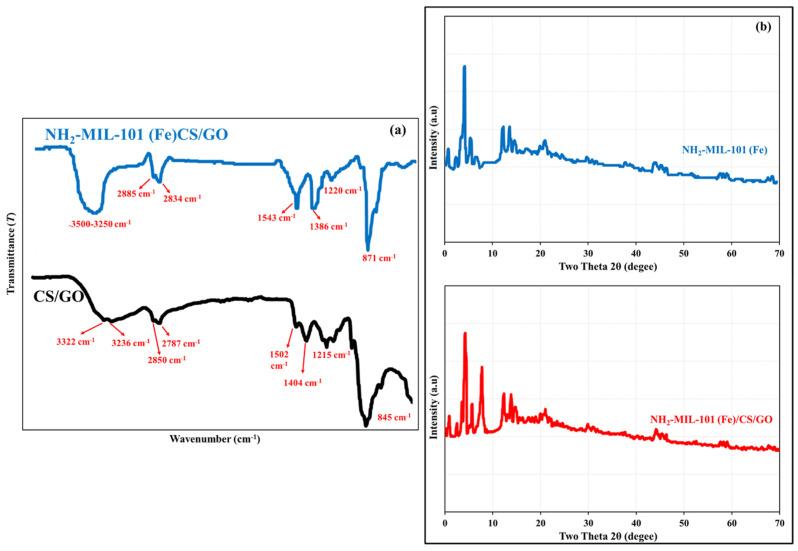
FTIR (**a**) and XRD (**b**) images of the NH_2_-MIL-101(Fe)/CS/GO.

The FTIR spectra of CS/GO, NH_2_-MIL-101(Fe) and NH_2_-MIL-101(Fe)/CS/GO nanocomposites displayed several characteristic peaks that highlight the contributions of all moieties in the matrix of the developed nanocomposite (Figure 2a). The O–H stretching vibrations of adsorbed water molecules and/or the H-bonded surface hydroxyl groups in the matrix of the nanocomposite are detected at 3500–3230 cm^−1^. The C–H stretching vibrational modes from aromatic and alkyl groups in CS and GO are detected at 2850–2885 and 2787–2834 cm^−1^, respectively, while the C–H bending in the -CH_3_ groups of CS is detected at 1386–1404 cm^−1^. The band at 1502–1543 cm^−1^ is specific to the asymmetric stretching of the coordinated carboxylate (COO^−^) groups of the organic linker, which confirms that the MIL-101 framework is intact. The N–H stretching vibrations, which are characteristic for -NH_2_ groups in CS, are detected at 3322 cm^−1^. The C–N stretching from the NH_2_ groups in the MOF (NH_2_-MIL-101(Fe)) is represented at 1215–1220 cm^−1^. The C–H bending vibrations of the methylene and methyl groups are represented by the sharp peak at 845–871 cm^−1^.

The XRD spectra of the NH_2_-MIL-101(Fe) and NH_2_-MIL-101(Fe)/CS/GO nanocomposites (Figure 2b) displayed sharp, well-defined diffraction peaks at 3.5°, 5.3°, 7.1°, 8.1°, 10.1°, and 11.5° [38,39,40], indicating the high crystallinity of amino-functionalized MOFs in the nanocomposites and even after the incorporation of CS and GO. This mainly confirms the well-defined porous structure with superior surface area and the availability of active sites of adsorption for MPs in the developed nanocomposite.

**Figure 3:**
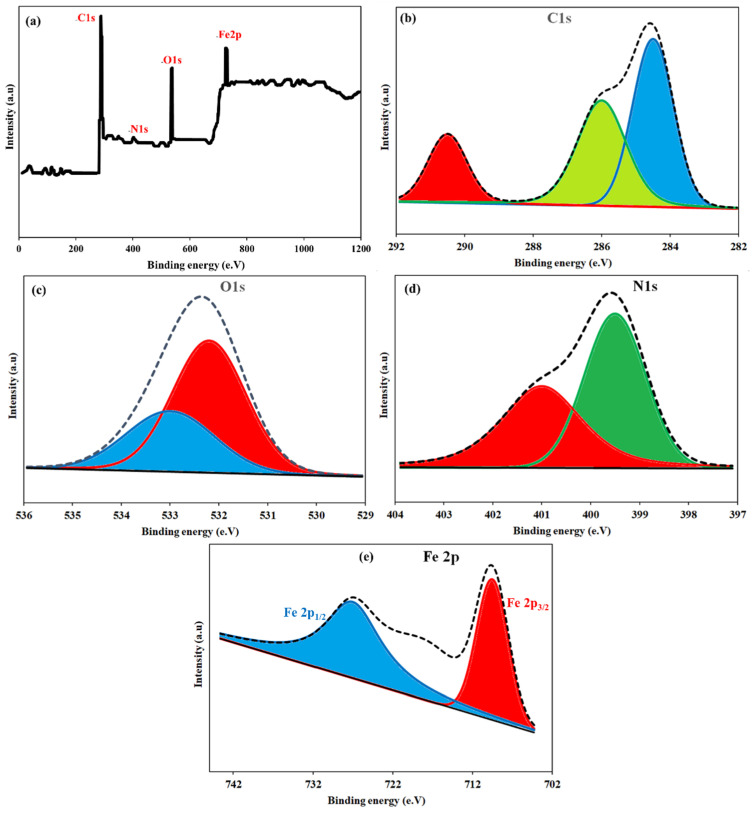
Full-spectrum (**a**) and high-resolution (**b**–**e**) XPS images of NH_2_-MIL-101(Fe)/CS/GO.

The XPS results strongly indicate the successful preparation of NH_2_-MIL-101(Fe)/CS/GO and detail the surface-specific interactions in both PET and PS adsorption. The survey spectrum showed the existence of C, N, O, and Fe elements, which was in line with the content of the composite (Figure 3a). In the high-resolution C1s spectrum (Figure 3b), the major component at ~284.6 eV is attributed to C–C/C=C bonds in the aromatic framework and in the GO sheets, the peaks at ~286 eV and ~288–289 eV are assigned to C–N/C–O and O–C=O groups, respectively [39,41]. The O1s spectrum is deconvoluted to be centered at ~531–533 eV, associated with metal–oxygen (Fe–O) and surface hydroxyl or carbonyl groups, and reveals a significant change after adsorption, suggesting a coordination between microplastic molecules and metal active sites (Figure 3c). The N1 profile (Figure 3d) is near ~399–401 eV, which can be attributed to the -NH_2_/-NH group of the MOF linker. In the region of the Fe 2p spectrum (Figure 3e), the typical Fe 2p_3_/_2_ and Fe 2p_1_/_2_ doublet representing Fe^3+^ in NH_2_-MIL-101 is maintained after loading; however, slight variation in peak shape and position after adsorption suggests the formation of a surface complex [38,42].

### 3.2. Effect of pH, Salinity, and Competitive Ions

**Figure 4:**
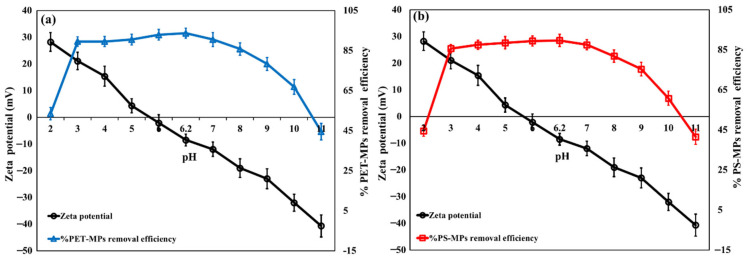
Zeta potential of the synthesized NH_2_-MIL-101(Fe)/CS/GO nanocomposite as a function of pH, and the corresponding effect of pH on the removal efficiency of PET, (**a**) and PS, (**b**) microplastics.

**Figure 5:**
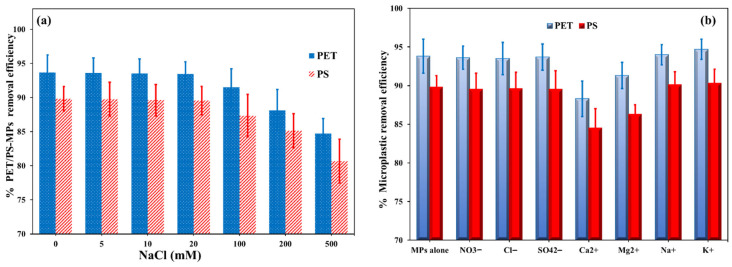
The impact of salinity (**a**) and competitive ions (**b**) on the PET/PS-MP removal efficiency by NH_2_-MIL-101(Fe)/CS/GO.

Since solution acidity strongly affects the surface charge of the adsorbent and, consequently, its interaction with microplastic particles, pH was considered a key adsorption parameter. As shown in Figure 4a,b, the removal efficiency of PET/PS by NH_2_-MIL-101(Fe)/CS/GO varies with pH and follows the change in zeta potential of the nanocomposite. The pHPZC of NH_2_-MIL-101(Fe)/CS/GO was 5.41, indicating that the surface is positively charged below this value and becomes negatively charged above it. In fact, the zeta potential decreased progressively from about +28.0 mV at pH 2, then shifted to negative values of about −3.0 mV at pH 5.0. The first highest removal efficiency (89.5% for PET and 85.7% for PS) was detected at pH 4.0. This is explained by the strong electrostatic interactions between the positively charged -NH_2_ groups (protonated below the pH_PZC_) of CS negatively charging PET/PS-MPs [43]. At pH greater than 4.0 (>4–6), the MPs’ removal efficiency is increased and reaches the second-highest removal efficiency (93.65% for PET and 89.71% for PS) at pH 6.2. One of the main processes influencing MP adsorption on carbon-based adsorbents at the neutral pH range is hydrophobic interaction [44]. Therefore, the adsorption of PET/PS-MPs on NH_2_-MIL-101(Fe)/CS/GO was facilitated by both hydrophobic and electrostatic interactions. The microplastic removal effectiveness steadily declined as the pH rose from 6.2 to 11. This was caused by increasing electrostatic repulsion at higher pH values because of the increased negatively charged surface of NH_2_-MIL-101(Fe)/CS/GO. Figure 5a illustrates how salinity affects PET/PS MP removal efficiency. Tests were conducted with NaCl concentrations ranging from 0 to 500 mM, imitating freshwater to seawater conditions. The MPs’ effectiveness for removal gradually reduced as the NaCl concentrations rose. Nevertheless, the removal efficiency dropped sharply to 100 mM NaCl after remaining comparatively constant (92.8%) up to 25 mM. According to the ANOVA results, PET/PS MP adsorption was significantly impacted by more than 100 mM (*p* < 0.01) of NaCl. This can be explained by the target MPs’ hydrodynamic diameter, which increased marginally at 0–25 mM NaCl but expanded significantly at 100 mM due to aggregation. Van der Waals forces predominated because the elevated Na^+^ concentrations caused PET/PS MPs to aggregate by electrostatic screening. Adsorption was significantly restricted by the PET/PS MPs’ aggregation at 500 mM. The clearance effectiveness stayed over 81.3% even while MPs aggregated at high salinities (100–500 mM). This prolonged performance may be explained by the strengthening of hydrophobic contacts between MPs and NH_2_-MIL-101(Fe)/CS/GO due to the dryness of their surface and compression of the electrical double layer [45].

Figure 5b illustrates the impact of the competing ions (Ca^2+^, Mg^2+^, Na^+^, K^+^, SO_4_^2−^, NO_3_^−^, and Cl^−^). When divalent cations, as compared with monovalent ions, especially Ca^2+^, were present, PET/PS-MP adsorption was greatly reduced. Divalent ions perform more effectively at collapsing the electrical double layer (EDL) [46], which improves instantaneous particle–particle aggregation and lowers the adsorption efficacy of MPs. Due to its greater ability [47] and bigger hydration radius (0.299 nm) [48], which restricted its direct contact with PET/PS MPs, Mg^2+^ showed weaker effects across the two divalent cations examined. Ca^2+^ more successfully adsorbed onto negatively charged PET/PS MP and GO/CS surfaces, significantly limiting adsorption, related to its smaller hydration radius (0.271 nm). In other words, adsorption was not significantly impacted by competing anions (Cl^−^ and SO_4_^2−^) (Figure 5b). Because PET/PS MPs have a negative surface charge, aggregation was mostly controlled by cations rather than anions. In contrast to Na^+^ and Mg^2+^, Ca^2+^ exhibited the most inhibitory effect on PET/PS-MP adsorption because of increased electrostatic screening and aggregation.

### 3.3. Reusability of NH_2_-MIL-101(Fe)/CS/GO

**Figure 6:**
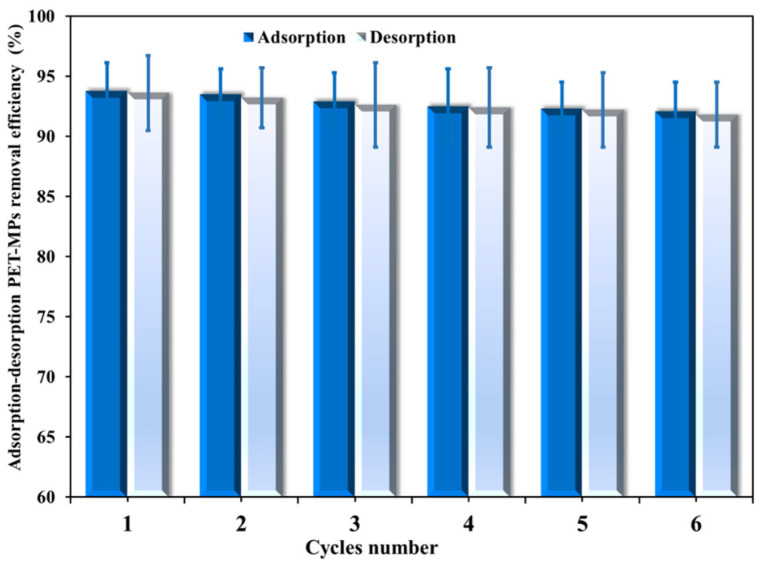
The recyclability of NH_2_-MIL-101(Fe)/CS/GO and PET/PS-MP removal efficiency at the optimum conditions.

When evaluating the practical and financial viability of adsorbents, reusability is a crucial consideration. To evaluate the recyclability of NH_2_-MIL-101(Fe)/CS/GO for PET-MP adsorption, adsorption–desorption experiments (Figure 6) conducted under equilibrium-condition regeneration showed that PET-MP removal efficiency by the NH_2_-MIL-101(Fe)/CS/GO nanocomposite was unaffected after the second cycle; however, it successfully maintained an average removal efficiency of over 92% during six consecutive cycles. However, a slight reduction in efficiency compared to the initial cycle was observed. This decrease is attributed to the occlusion of active sites by MPs via chemisorption, which impacts its effectiveness in subsequent cycles [49]. Therefore, the developed NH_2_-MIL-101(Fe)/CS/GO can be reused multiple times.

### 3.4. Impact of Time and Kinetic Study

**Figure 7:**
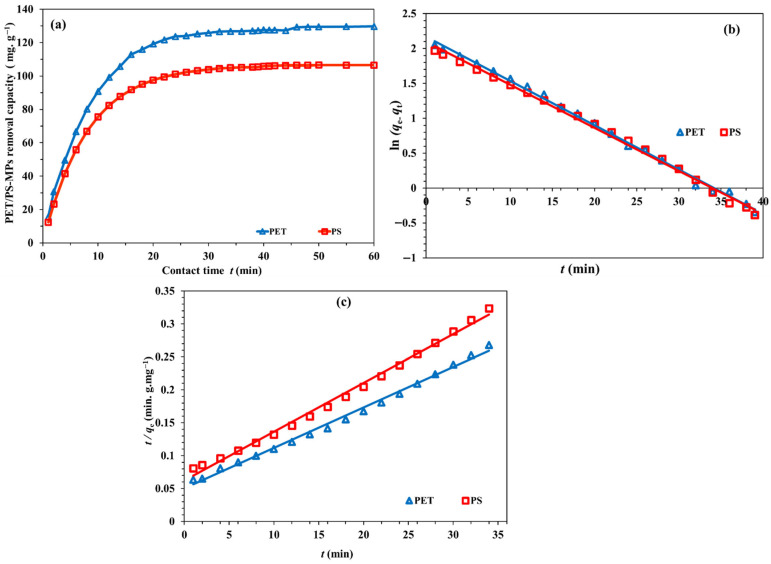
Impact of contact time (**a**), PFO (**b**) and PSO (**c**) for the PET/PS-MP adsorptive removal of NH_2_-MIL-101(Fe)/CS/GO.

The sorption of kinetic processes has significance as they correlate the rate of solute intake to the reaction pathways. For the goal of designing a full-scale batch adsorption process, sorption kinetic models might effectively examine the mechanisms governing the sorption process. Figure 7a shows the effects of time for the adsorption of PET and PS microplastics by NH_2_-MIL-101(Fe)/CS/GO under the optimal conditions. The removal capacities of PET/PS-MPs are illustrated by a rapid sorption kinetic rate during the first 10 min, which is followed by an incremental rise in the MPs’ adsorption rate until equilibrium is reached in 40 min. As shown in Figure 7b,c, two kinetic models, pseudo-first-order (PFO) and pseudo-second-order (PSO), were used to comprehend the kinetic regime. Table 2 contains the linear equations for the two models along with their fitting parameters. Both kinetic models described the adsorption data for PET and PS reasonably well, with high correlation coefficients in all cases. For PET, the R^2^ values were 0.9968 for the PFO model and 0.9944 for the PSO model, while for PS they were 0.9956 and 0.9942, respectively. Because these values are very close, model evaluation based only on R^2^ may be misleading. To obtain a more reliable comparison, additional error functions, including RMSE and χ^2^, were considered in the revised manuscript. For PET, the PSO model gave lower error values (RMSE ≈ 2.8 mg g^−1^; χ^2^ ≈ 1.9) than the PFO model (RMSE ≈ 5.5 mg g^−1^; χ^2^ ≈ 4.7). A similar trend was found for PS, where the PSO model produced RMSE ≈ 2.1 mg g^−1^ and χ^2^ ≈ 1.4, compared with RMSE ≈ 5.2 mg g^−1^ and χ^2^ ≈ 4.3 for the PFO model. In addition, the equilibrium adsorption capacities calculated from the PSO model were closer to the experimental values, indicating that this model represents the kinetic data more satisfactorily.

Taken together, these results suggest that the adsorption process is better described by the PSO model, although the good fit of both models indicates that the overall uptake may involve more than one type of interaction. This behavior is consistent with the multifunctional surface of NH_2_-MIL-101(Fe)/CS/GO, where both hydrophilic and hydrophobic interactions can contribute to PET and PS removal. The similarity of the kinetic constants and calculated capacities for both microplastics also implies that the available binding sites on the nanocomposite interact with PET and PS in a broadly comparable manner.

### 3.5. PET/PS-MP Sorption Isotherms

The sorption isotherm could be used to assess various ways that a particular analyte interacts with any sorbent in equilibrium. Freundlich and Langmuir isotherm models were used to confirm the experimental results for the sorption of PET and PS microplastic by the NH_2_-MIL-101(Fe)/CS/GO [52,53]. Langmuir suggested that monolayer sorption would form non-homogeneous surfaces, but Freundlich’s model suggested that multilayer sorption would form. The sorption capacity of NH_2_-MIL-101(Fe)/CS/GO was assessed using the sorption isotherms of the target microplastics (1–50 mg L^−1^). The Langmuir and Freundlich linear adsorption models were used separately to examine the equilibrium adsorption isotherm data. The fitting parameters of the two models were compared and compiled in Table 3. The isothermal sorption data for PET and PS sorption isotherms fit well with both the Langmuir and Freundlich models. The excellent fit for both models implies that the adsorption process could be a combination of both types or that the system’s behavior is more complex and can be described by both approaches. For PET and PS-MPs, the highest sorption capacities were determined to be 255 and 321 mg g^−1^, respectively. PET/PS adsorption is advantageous according to the sorption characteristics. When evaluating the preferred adsorption of the targeted MPs and the NH_2_-MIL-101(Fe)/CS/GO sorbent, the adsorption intensity n for the Freundlich plots is higher (n > 1) than one. The adsorption affinity between PET/PS-MP ions and NH_2_-MIL-101(Fe)/CS/GO is favorable, as indicated by the Langmuir dimensionless separation factor (*R*_L_) value being less than unity (0 < *R*_L_ (0.01–0.06) < 1).

### 3.6. Synergistic Roles of CS, GO, and NH_2_-MIL-101(Fe) in PET/PS Microplastic Adsorption by NH_2_-MIL-101(Fe)/CS/GO and Possible PET/PS-MP Adsorption Mechanism

A comparative adsorption study was carried out to clarify the role of each component in the developed NH_2_-MIL-101(Fe)/CS/GO nanocomposite. The results show that the high removal performance of the composite arises from the cooperative action of GO, CS, and NH_2_-MIL-101(Fe), rather than from the contribution of any single component alone. Pristine GO exhibited the lowest removal efficiency, reaching only 40.1 ± 2.5% for PET and 37.5 ± 1.8% for PS. This limited performance can be attributed to the fact that GO provides high-surface-area support and contributes π-rich domains, besides the hydrogen-bonding and outer-sphere interactions with microplastic surfaces.

When CS was introduced, the removal efficiency improved, particularly for PS, reaching 41.9 ± 3.1% for PET and 74.5 ± 2.3% for PS. This improvement highlights the importance of the amino-rich chitosan matrix, which can strengthen adsorption through electrostatic attraction and hydrogen-bonding interactions. The best removal rates were achieved when NH_2_-MIL-101(Fe), CS, and GO were combined into one system. The composite removed 93.8 ± 3.5% of PET and 89.8 ± 3.3% of PS. The NH_2_-MIL-101(Fe) moiety supplies additional porous adsorption sites and functional groups that promote stronger binding. These results clearly confirm a strong synergistic effect among the three components, and the combination of these features creates a multifunctional interface that favors efficient capture of both PET and PS microplastics. The enhanced adsorption behavior of NH_2_-MIL-101(Fe)/CS/GO is likely governed by several complementary mechanisms operating at the same time.

Based on the impact of the pH batch, kinetic and reusability studies, the removal of PET and PS microplastics using the NH_2_-MIL-101(Fe)/CS/GO nanocomposite is mainly attributed to hydrophobic and hydrophilic interactions. The remarkable adsorption efficiency is probably largely due to the hydrophobic interactions between the GO/CS matrix and the nonpolar surfaces of PET and PS. Additionally, CS and Fe-MOF’s amino functional groups may promote adsorption at the interface by facilitating electrostatic forces with the negative-charged microplastic surfaces. In addition, the binding of PET/PS-MPs may be strengthened by the π-π stacking interactions that occur between GO sheets and the aromatic rings of PS and PET. The interaction between the PS benzene rings and Fe clusters in NH_2_-MIL (Fe)-101, as well as the polar attraction between Fe-MOF aromatic oxygen atoms and C-H bonds in the PET/PS chain, is mainly van der Waals forces [53]. This confirms that the functionalization of CS/GO with NH_2_-MIL (Fe)-101 relates covalent and non-covalent interactions in a synergetic manner. The typical adsorption capacity of PET (94%) and PS (90%) at equilibrium time (40 min) indicates that the nanocomposite material offers these microplastics a robust and stable interaction surface, probably via the combination of H-bonding, van der Waals forces, and electrostatic attractions, enabling their efficient removal from aqueous solutions.

### 3.7. Application of NH_2_-MIL-101(Fe)/CS/GO for PET/PS-MP Removal from Different Environmental Samples

**Figure 8:**
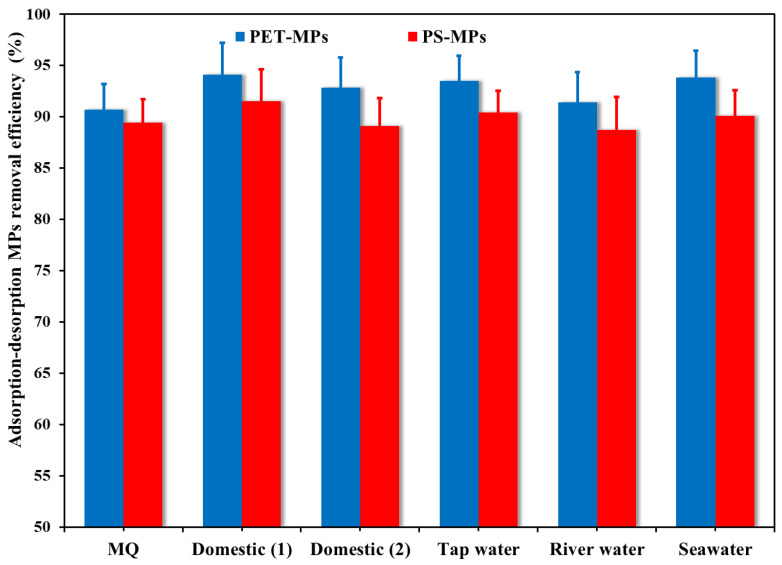
PET/PS-MP (Co = 15 mg L^−1^) removal efficiency by NH_2_-MIL-101(Fe)/CS/GO nanocomposite from different environmental samples with different matrices.

The effects of environmental and wastewater samples (two domestic wastewater, tap, river, and seawater samples) on microplastic removal efficiency and their water quality parameters are displayed in Figure 8, respectively. The pH of the different sentimental samples (7.22–8.31) is comparable, and this excludes the impact of pH from the interpretation of results. Based on the water quality parameters that are depicted in Table 1, the difference in PET-MP removal efficiency between the water samples under study might be based on several factors. The highest PET-MP removal efficiency was obtained with domestic water sample 2 (94.1%) and seawater (93.8%), followed by tap water (93.5%), river water (91.4%) and MQ water (90.7%). The difference in MP removal efficiency between river water and the other samples may be due to the presence of dissolved organic matter, as the river water sample exhibited the lowest TDS value (125.7 mg L^−1^), as depicted in Table 1. Natural dissolved organic matter (NDOM) can interfere with MP adsorption by competing for active sites on NH_2_-MIL-101(Fe)/CS/GO [43,54] and by adsorbing onto PET-MPs, altering their surface properties [55]. Tap water showed a higher microplastic removal capacity than MQ water, and this was mainly attributed to the low content of Na^+^ ion (Na^+^ = 6.8 mg L^−1^) concentrations partially neutralizing electrostatic repulsion between negatively charged PET-MPs and NH_2_-MIL-101(Fe)/CS/GO via charge screening.

High amounts of the competing ions (K^+^, Na^+^, Ca^2+^, Mg^2+^, SO_4_^2−^, NO_3_^−^, and Cl^−^) coexist with dissolved organic matter in seawater. Despite high concentrations of Ca^2+^ and Mg^2+^ alone preventing PET-MP adsorption by causing aggregation, their combination with NDOM in seawater may improve PET-MP adsorption onto NH_2_-MIL-101(Fe)/CS/GO through an MP stabilization mechanism mediated by NDOM. Instead of developing NDOM–metal complexes with low concentrations (15 mg L^−1^) of PET-MPs, the low-molecular-mass NDOM with negative charges in seawater quickly bonds with copious Ca^2+^ (512.95 mg L^−1^) and Mg^2+^ (1274.4 mg L^−1^). These complexes inhibit aggregation by partially adhering to the surfaces of PET-MPs. Other investigations have documented the synergistic effects of Mg^2+^ and NDOM through complex formation, which facilitates MP transport by preserving colloidal stability [56,57]. Additionally, complex-stabilized PET-MPs and the NH_2_-MIL-101(Fe)/CS/GO surface interact more hydrophobically due to the high ionic strength of saltwater. This implies that the effective removal of MPs may be impacted by the complex matrix of seawater, specifically the synergistic effects of NDOM and divalent cations. Notably, the developed nanocomposite’s ability to remove MPs and even MPs and NPs in marine environments is demonstrated by the highest MP removal efficiency in seawater, which is encouraging for practical applications.

The statistical evaluation of the data presented in Figure 8 was performed using one-way ANOVA to assess the significance of differences between experimental conditions. The analysis yielded *p*-values greater than 0.05 for all tested variables, indicating that the variations in removal efficiency are statistically insignificant at the 95% confidence level. Furthermore, the calculated F-values were consistently lower than the corresponding F-critical values, confirming the absence of significant differences among the groups. This statistical outcome corroborates the visual similarity observed in Figure 8 and suggests that the adsorption process remains consistent across the studied conditions.

## 4. Conclusions

This study revealed that the fabricated NH_2_-MIL-101(Fe)/CS/GO composite performed as an effective adsorbent for removing PET/PS microplastics, which are increasingly concerned pollutants in aquatic environments. The sorption process was mainly influenced via pore-filling and physisorption/chemisorption mechanisms, driven by hydrophobic interactions, electrostatic forces, Van der Waals forces, H-bonding, and π–π interactions. Water quality elements like interfering ions, pH, and ionic strength influenced the sorption of PET/PS-MPs by promoting aggregation and modifying the surface chemistry of the PET/PS-MPs and the NH_2_-MIL-101(Fe)/CS/GO materials. The best removal efficiencies for PET/PS-MPs with NH_2_-MIL-101(Fe)/CS/GO were 90–93% at a pH of 6.2, with a 40 min equilibrium time for microplastics in the particle size range of 25–30 μm. Notably, high PET/PS-MP removal efficiencies were observed in domestic wastewater and seawater (92–95%), demonstrating the potential of the NH_2_-MIL-101(Fe)/CS/GO nanocomposite for the removal of PET/PS-MPs in aquatic ecosystems in different matrices. The developed nanocomposite also demonstrated excellent reusability after six cycles with less than 3.0% of the initial MP removal efficiency. These results indicate that the NH_2_-MIL-101(Fe)/CS/GO nanocomposite has extensive applicability for mitigating microplastic pollution, with successful implementation in wastewater and marine environments.

## Figures and Tables

**Table 1:** Water quality parameters of different environmental samples used for PET/PS-MP remediation by NH_2_-MIL-101(Fe)/CS/GO nanocomposite.

Parameter	Domestic Wastewater (1)	Domestic Wastewater (2)	Tap Water	River Water	Seawater
pH	7.22 ± 0.05	7.85 ± 0.03	7.15 ± 0.08	7.22 ± 0.05	8.31 ± 0.02
TDS (mg L^−1^)	917 ± 96	965 ± 105	284 ± 75	126 ± 43	34,861 ± 153
Na^+^ (mg L^−1^)	39.51 ± 4.35	44.62 ± 2.17	6.14 ± 0.75	5.35 ± 0.81	10,016 ± 257
K^+^ (mg L^−1^)	18.22 ± 5.1	13.41 ± 2.2	1.8 ± 0.03	3.14 ± 1.4	394 ± 32
Ca^2+^ (mg L^−1^)	35.11 ± 9.5	42.71 ± 6.3	7.18 ± 2.7	11.45 ± 2.5	513 ± 73
Mg^2+^ (mg L^−1^)	64.7 ± 1.5	71.5 ± 1.7	1.83 ± 0.08	4.15 ± 0.87	1274 ± 315
Cl^−^ (mg L^−1^)	2.19 ± 0.02	1.53 ± 0.05	11.52 ± 2.5	6.33 ± 1.41	19,352 ± 410
NO_3_^−^ (mg L^−1^)	1.52 ± 0.07	5.3 ± 1.1	4.93 ± 1.22	11.50 ± 1.53	ND
SO_4_^2−^ (mg L^−1^)	16.43 ± 3.32	15.91 ± 3.21	6.81 ± 1.52	8.13 ± 1.55	2518.5 ± 357

**Table 2:** Linear kinetic equations for the adsorption of PET and PS on NH_2_-MIL-101(Fe)/CS/GO nanocomposite at 25.0 °C, pH 6.2 and 300 mg mass dose [50,51].

Kinetic Model	Linear Kinetic Equation	Calculated Parameters	PET	PS
PFO	$\ln\;(q_{e}-q_{t})=\ln\;q_{e}-k_{1}t$	*q*_e_ (mg g^−1^)	147.9	124.5
		*k*_1_ (min^−1^)	0.146	0.142
		*R* ^2^	0.9968	0.9956
PSO	$\frac{t}{q_{t}}=\;\frac{1}{k_{2}\;q_{e}^{2}}+\frac{t}{q_{e}}$	*q*_e_ (mg g^−1^)	161.3	135.1
		*k*_2_ (g mg^−1^ min^−1^)	5.2 × 10^5^	2.9 × 10^5^
		*R* ^2^	0.9944	0.9942

*q*_e_ and *q*_t_ (mg g^−1^) are the equilibrium adsorption capacity at equilibrium and at time *t*, respectively. *C*_e_ is the PET or PS concentration at equilibrium. *K*_1_ and *K*_2_ are the first- and second-order adsorption rate constants of kinetic models.

**Table 3:** Linear Langmuir and Freundlich equilibrium fitting parameters for adsorption of PET and PS microplastics onto NH_2_-MIL-101(Fe)/CS/GO nanocomposite.

Isotherm Model and Parameters	PET	PS
*Langmuir model:* $\frac{C_{e}}{q_{e}}=\frac{1}{\left(K_{L}\;q_{m}\right)}+\frac{C_{e}}{q_{m}}$		
*q*_m_ (mg g^−1^)	321.4	255.1
*K*_L_ (Lm g^−1^)	0.0647	0.0272
*R* ^2^	0.9971	0.9963
*R_L_*	0.01 > *R*_L_ > 0.05	0.01 > *R*_L_ > 0.06
*Freundlich model:* $\ln q_{e}=\ln K_{F}+\frac{1}{n}\ln C_{e}$		
*K*_F_ (mg^1−n^·L^n^·g^−1^)	92.51	75.36
*n*	1.43	2.188
*R* ^2^	0.9905	0.9914

## Data Availability

The original contributions presented in this study are included in the article. Further inquiries can be directed to the corresponding author.
